# Antioxidant Activity of New Sulphur- and Selenium-Containing Analogues of Potassium Phenosan against H_2_O_2_-Induced Cytotoxicity in Tumour Cells

**DOI:** 10.3390/cimb44070216

**Published:** 2022-07-07

**Authors:** Lyubov S. Klyushova, Natalya V. Kandalintseva, Alevtina Y. Grishanova

**Affiliations:** 1 Institute of Molecular Biology and Biophysics, Federal Research Center of Fundamental and Translational Medicine, 2/12 Timakova Str., 630060 Novosibirsk, Russia; agrish@niimbb.ru; 2 Department of Chemistry, Novosibirsk State Pedagogical University, 28 Vilyuyskaya Str., 630126 Novosibirsk, Russia; iesen@nspu.ru

**Keywords:** phenolic antioxidants, potassium phenosan, sulphur, selenium, in vitro research, cytotoxicity, cytostaticity, antioxidant activity, hydrogen peroxide

## Abstract

Among known phenolic antioxidants, the overwhelming majority of compounds have lipophilic properties and the number of known water-soluble compounds is very small. The list of hydrophilic phenolic antioxidants can be expanded via the synthesis of a structurally related series of polyfunctional compounds for further research on their biological activity in vitro. New sulphur- and selenium-containing analogues of antioxidant potassium phenosan were synthesised. In vitro cytotoxicity and cytostaticity as well as antioxidant activity against H_2_O_2_-induced cytotoxicity to human cell lines (HepG2, Hep-2 and MCF-7) were investigated by high-content analysis. A selenium-containing analogue showed higher biological activity than did a sulphur-containing one. As compared to the activity of potassium phenosan, the selenium-containing analogue had a cell line-dependent antioxidant effect against H_2_O_2_-induced cytotoxicity: comparable in HepG2 cells and greater in Hep-2 cells. The selenium-containing analogue significantly increased the death of MCF-7 cells at concentrations above 50 µM. The sulphur-containing analogue has lower biological activity as compared to potassium phenosan and the selenium-containing analogue.

## 1. Introduction

The use of natural or synthetic antioxidants for the prevention and treatment of diseases involving oxidative stress is a pathogenetically sound strategy [1]. Nonetheless, numerous randomised controlled trials have yielded contradictory results regarding effectiveness of their application to anticancer therapy (primarily such vitamins as A, E and C). The results of such studies can be divided into three groups: one of which indicates a beneficial effect of increased consumption of nutrient antioxidants, the second group indicates a lack of effectiveness, and the third one a negative effect on health [2,3,4]. In some preclinical studies, antioxidants have been found to promote tumour growth and metastasis [5,6]. To summarise, antioxidants have distinct effects on different stages of tumorigenesis in a tumour type-specific fashion.

Genetic changes enable cancer cell survival at high reactive oxygen species (ROS) levels by raising their antioxidant status [7].Preclinical studies suggest that an increase in ROS concentrations in cancer cells through ROS-inducing therapies can be an effective anticancer strategy [8]. Antioxidants may also enhance cytotoxic effects of anti-neoplastic drugs, reduce their toxicity towards normal cells [9], and serve as anti-tumour agents themselves [10]. This situation, usually explained by a natural concentration-dependent inversion of the antioxidant effect (conversion into a pro-oxidant one) [11,12,13], makes relevant the research interest in synthetic inhibitors of free-radical processes as prospective antioxidant therapies.

Among synthetic inhibitors of free-radical-mediated oxidation, some of the most effective compounds are alkylated phenols containing functional groups with bivalent sulphur in substituents [14,15]. The high effectiveness of such thioalkylphenols is due to the synergistic combination of the anti-radical activity of their hydroxyaryl moieties and the anti-hydrogen peroxide activity of sulphur-containing groups. Lipophilic derivatives of sulphur-containing phenolic antioxidants that are described in the literature and used practically as antioxidants are numerous and structurally diverse [16,17,18]. Earlier, extensive studies have been carried out regarding patterns of changes in the inhibitory activity of such antioxidants depending on their structure [18,19]. The results have made it possible to perform targeted synthesis of compounds with high antioxidant activity and pronounced bioantioxidant properties: they effectively inhibit lipid peroxidation in vitro, block the development of oxidative stress in vivo, exert protective action against various free-radical-associated pathologies and are promising leads for the creation of new antioxidant drugs.

Selenium- and tellurium-containing alkylphenols apparently also possess valuable bioantioxidant properties [20,21,22,23,24]; however, such compounds have been studied insufficiently, and to the best of our knowledge, the literature contains data on a relatively small number of classes of exclusively lipophilic compounds.

At the same time, hydrophilic antioxidants are more attractive for use in biology, veterinary medicine, and medicine and are characterised by high bioavailability, rapid biological transport, convenience of administration and dosing methods, and suitability for infusion therapy of acute pathologies [13].

Traditional applications of phenolic antioxidants have not required hydrophilic properties; on the contrary, lipophilicity has been preferred. In this regard, it is not surprising that the number of known water-soluble derivatives of phenolic antioxidants is very limited (water-soluble tocopherol derivatives such as Trolox, phenolic acids derivatives such as sodium or potassium salt of phenosan acid and others) [25,26,27,28,29,30]. Such antioxidants are created according to a single principle and consist of an alkylphenol backbone and a polar (usually ionogenic) moiety, which makes the compound soluble in aqueous media. The overwhelming majority of these derivatives are monofunctional antioxidants and inhibit an oxidative process only through interaction with active radicals. Hydrophilic sulphur-containing phenolic antioxidants, for which a higher antioxidant activity should be expected as compared to no-sulphur parent chemicals (just as in lipophilic antioxidants), are represented only by a few compounds [31]. They significantly differ in their structure, and the data on their antioxidant activity are fragmentary. Meanwhile, some comparative studies on antioxidant properties of hydrophilic antioxidants of various structures indicate that the ‘structure–antioxidant activity’ relations previously identified for their lipophilic analogues, are hardly applicable [32,33]. Thus, until recently, there have been no opportunities for targeted synthesis of new hydrophilic antioxidants that are capable of inhibiting oxidative processes in biological systems more effectively than the existing analogues can.

In light of the above, it seemed relevant to expand the list of hydrophilic phenolic antioxidants by the synthesis of structurally related polyfunctional compounds and to study their biological activity in vitro.

In this work, we synthesised new sulphur- and selenium-containing analogues of an antioxidant called potassium phenosan and then examined their potential cytotoxic and antioxidant effects in an in vitro model.

## 2. Materials and Methods

### 2.1. Synthetic Chemistry

Commercially available chemicals and solvents ((Sigma-Aldrich (Darmstadt, Germany), and Reachim (Moscow, Russia)) were used. The solvents were purified and dried before use according to standard procedures [34]. Potassium phenosan [**PP**, potassium 3-(3,5-di-tert-butyl-4-hydroxyphenyl)propionate], 3-[3-(3,5-di-tert-butyl-4-hydroxyphenyl)propylseleno]propanoic acid (**1a**), and potassium 3-[3-(3,5-di-tert-butyl-4-hydroxyphenyl)propylseleno]propionate (**1b**) weresynthesised by a procedure described previously [35]. NMR spectra are given in Figures S1–S3.

#### 2.1.1. Synthesis of 2-(3,5-Dimethyl-4-hydroxybenzylthio)propanoic Acid (**2a**)

2,6-Dimethylphenol (44 mmol, 5.35 g) and 3-thiopropanoic acid (42 mmol, 4.42 g) were dissolved separately in ethanol (10 mL). The resulting solutions were combined, and the mixture was added drop-wise to a solution of KOH (63.5 mmol, 4.24 g) in ethanol (10 mL) under argon atmosphere. The mixture was stirred at room temperature until KOH dissolved completely. Then, formalin (131 mmol, 9.8 mL) was added, and the resulting solution was boiled for 1 h. The mixture was cooled, 40 mL of a saturated NaHCO_3_ solution was added, and the resultant solution was mixed and incubated with 25 mL of toluene. The aqueous fraction was cooled on ice and acidified with HCl to pH 2–3. The precipitated crystals were filtered off, washed with ice water, and dried. The yield was 9.17 g (92%). Colourless crystals, m.p. 72–73 °C. Elemental analysis: Found (%): C, 60.06; H, 6.78; S, 13.27. C_12_H_16_O_3_S; Calcd (%): C, 59.97; H, 6.71; S, 13.34. UV: λ_max_ (EtOH, nm) 278, lg ε (L·M^−^^1^·cm^−^^1^) 3.18. IR spectra (ν, cm^−^^1^): 3607 (OH), 3505 (OH), 1715 (C=O). ^1^H-NMR (600 MHz, CDCl_3_) δ: 2.20 (s, 6H, Me), 2.54 (m, 2H, CH_2_CH_2_COOH), 2.58 (m, 2H, CH_2_COOH), 3.56 (s, 2H, ArCH_2_), 5.3 (s, 1H, ArOH), 6.84 (s, 2H, H2andH6), 11.1 (s, 1H, COOH). ^1^H-NMR spectrumis shown in Figure S4.

#### 2.1.2. Synthesis of Potassium 3-[4-Hydroxy-3,5-dimethylbenzylthio]propanoate (**2b**)

KHCO_3_ (7.43 mol, 0.74 g) and H_2_O (25 mL) were added to a solution of 2-(3,5-dimethyl-4-hydroxybenzylthio)propanoic acid (7.5 mmol, 1.8 g) in ethanol (7.5 mL). The mixture was heated in an argon atmosphere and refluxed for 1.5 h, and next, the solvent was distilled off. The residue was air dried, crushed, washed with toluene, and re-dried. The yield was 2.05 g (99%). Colourless crystals, m.p. 173–176 °C. Elemental analysis: Found (%): C, 51.85; H, 5.38; S, 11.63. C_12_H_15_KO_3_S; Calcd (%): C, 51.77; H, 5.43; S, 11.63. UV: λ_max_ (EtOH, nm) 278, lg ε (L·M^−^^1^·cm^−^^1^) 3.18. ^1^H-NMR (600 MHz, D_2_O) δ: 2.04 (s, 6H, Me), 2.29 (t, *J* = 7.5 Hz, 2H, CH_2_CH_2_COOK), 2.49 (t, *J* = 7.5 Hz, 2H, CH_2_COOK), 3.48 (s, 2H, ArCH_2_), 6.83 (s, 2H, H2 and H6). ^13^C-NMR (150 MHz, D_2_O) δ: 15.41 (CH_3_), 27.07 (CH_2_), 34.25 (CH_2_), 37.00 (ArCH_2_), 125.36 (C3, C5), 128.74 (C2, C6), 130.41 (C1), 150.46 (C4), 180.73 (COOK). NMR spectra (^1^H and ^13^C) are given in Figures S5 and S6.

### 2.2. Equipment

NMR spectra were recorded on a BrukerAvance 600 spectrometer (Bruker Biospin, Rheinstetten, Germany) [600.13 MHz (^1^H) and 150.90 MHz (^13^C-{^1^H}] in D_2_O. Infrared (IR) spectra were acquired on a Vektor 22 Fourier spectrometer (Bruker Optic, Ettlingen, Germany) in KBr (150:1). UV spectra were recorded by means of a Shimadzu UV-1800 instrument (Shimadzu Europa GmbH, Duisburg, Germany) for the solutions in EtOH (c = 4 × 10^−^^5^ M⋅L^−^^1^). Melting points were measured by a capillary method (at a heating rate of 0.5 deg⋅min^−^^1^) using a MP 50 Mettler Toledo apparatus. Absorbance at 570 nm was measured on an ELISA reader (Tecan, GENiosELIASA Co., Salzburg, Austria). The UV absorption spectra for the antioxidant capacity assays were recorded in a water-ethanol (4:1) solution on an Agilent 8453 UV-visible spectrophotometer (Agilent Technologies Deutschland GmbH, Waldbronn, Germany) using cuvettes with a 1 cm light path.

### 2.3. Cell Culture

Biological research was performed on human cell lines HepG2 (hepatocellular carcinoma), Hep-2 (larynx carcinoma) and MCF-7 (breast adenocarcinoma). The cell lines were purchased from the State Research Center of Virology and Biotechnology VECTOR (Novosibirsk, Russia) and cultured in Iscove’s Modified Dulbecco’s Medium (IMDM, pH 7.4) supplemented with 10% of foetal bovine serum (FBS) in a humidified atmosphere (5% CO_2_ and 95% air) at 37 °C.

### 2.4. In Vitro Cytotoxicity and Cytostaticity Assays

Cell viability was detected by Hoechst 33342/propidium iodide (PI) staining as previously described [36]. HepG2, Hep-2 and MCF-7 cells were seeded in 96-well plates at 5 × 10^3^ cells per well and cultured for 24 h. Then, the cells were treated for 48 h with a tested compound dissolved in ethanol, in a 50% aqueous solution of ethanol, or in water at concentrations 1–10^4^ μM. The treated cells and control cells were stained with a mixture of fluorescent dyes (Hoechst 33,342 (Sigma-Aldrich, Buchs, Switzerland) and PI (Invitrogen, Inchinnan, UK)) for 30 min at 37 °C. An IN Cell Analyzer 2200 (GE Healthcare, Chalfont Saint Giles, UK) was used for automatic imaging of four visual fields per well at 200× magnification in bright-field and fluorescence channels. In accordance with morphological changes, the cells were classified by means of the IN Cell Investigator image analysis software (GE Healthcare, UK) as live cells (normal nuclei: non-condensed chromatin uniformly dispersed throughout the nucleus), apoptotic cells (round cells with bright chromatin that is highly condensed or fragmented) and dead cells (primarily stained with PI owing to impaired permeability of the plasma membrane: enlarged nuclei with smooth normal structure or slightly condensed nuclei). The cytotoxic activity was determined as half-maximal lethal concentration (LC_50_), which was defined as ‘the compound concentration that reduces the number of live cells by 50%’ and was calculated from curves constructed by plotting cell survival (%) versus drug concentration (µM). The cytostatic activity was determined as half-maximal inhibitory concentration (IC_50_), which was calculated from a curve constructed by plotting a cell count (%) versus drug concentration (µM).

### 2.5. The Antioxidant Activity Assay

#### 2.5.1. Ferric-Ion-Based TAC Assays

The total antioxidant capacity was determined by the 1,10-phenanthroline (o-phen) method with incubation as described previously [37]. Briefly, the o-phen-Fe(III) reagent was prepared by mixing 0.198 g of o-phen, 2 mL of 1 M HCl and 0.16 g of NH_4_Fe(SO_4_)_2_·12H_2_O followed by dilution with water to 100 mL. The reaction mixture [80 µL of the 1,10-phen reagent solution, 380 µL of EtOH (96%), 1.52 mL of H_2_O and various concentrations of a tested antioxidant (20 μL)] was incubated at 50 °C for 30 min. Absorbance was detected at 510 nm. The results of triplicate assays (TAC values) were expressed in µmol of Fe(II) equivalents and were calculated from a calibration curve built by means of ferrous sulphate (FeSO_4_·7H_2_O) standards (20–100 μM).

#### 2.5.2. The DPPH˙ Radical-Scavenging Assay

This assay was carried out by a previously reported method [38] with some modifications. The solution of DPPH˙ (2,2-di[4-tertoctylphenyl]-1-picrylhydrazyl) radical in EtOH (0.1 mM, 2 mL) was mixed with solution of a tested compound in ethanol (2–20 μM, 20 μL). The reduction of DPPH˙ was monitored as absorbance at 517 nm every 5 min for 30 min. The absorbance of a blank solution of DPPH˙ (2 mL) as a control was also registered at 517 nm.

#### 2.5.3. The ABTS˙^+^ Radical-Scavenging Assay

This assay was performed according to a previously described method [38]. In short, 1.0 g of MnO_2_ was added to a solution of ABTS [2,2′-azino-bis-(3-ethylbenzothiazoline-6-sulphonic acid)] in phosphate buffer (54.2 mg, 10 mL), and the reaction mixture was shaken. After 30 min, the solution was centrifuged for 5 min and filtered. The filtrate was diluted with phosphate buffer (absorbance 0.70 ± 0.01 at 723 nm reached). Different concentrations (1–10 μM) of a tested compound (20 μL) were added to 2 mL of the ABTS˙^+^ solution and incubated for 10 min at room temperature. The decrease in absorbance was monitored at 734 nm.

For all assays, radical-scavenging activity *(RSA)* was calculated using the following Equation:

(1) $$RSA\;\left(\%\right)=\left(1-\frac{A}{A_{0}}\right)\times 100,$$

where *A*_0_ is the absorbance of the control, and *A* is the absorbance of the test sample.

The concentration of a compound reducing radical concentration by 50% (IC_50_) was calculated from a curve constructed by plotting *RSA* (%) versus drug concentration (µM).

#### 2.5.4. Hydrogen Peroxide (H_2_O_2_)-Induced Oxidative Stress and Evaluation of Cell Survival

H_2_O_2_ was used for induction of oxidative stress [39]. Cells were seeded in 96-well plates (5 × 10^3^ cells per well) in IMDM containing 10% of FBS and incubated at 37 °C in a humidified atmosphere containing 5% of CO_2_. After overnight cultivation, they were treated with different concentrations (0.1–8.0 mM) of H_2_O_2_, freshly prepared from a 3% stock solution, with untreated cells serving as a control. At the end of the 4 h incubation at 37 °C and 5% CO_2_, the Hoechst 33342/PI assay was carried out. The percentage of viable cells was calculated for each cell line, and the concentrations of H_2_O_2_ that caused ~20% and 40% cell death were used for induction of oxidative stress. The chosen H_2_O_2_ concentrations were 0.9 and 1.5 mM for HepG2 cells, 1.5 and 3 mM for MCF-7 cells, and 2 and 4 mM for Hep-2 cells. To assess in vitro the antioxidant activity against H_2_O_2_ cytotoxicity, the cells were seeded in 96-well plates, and one of the tested compounds was added to the cell cultures at concentrations 1–75 μM as described above. After 24 h, the cells were exposed to H_2_O_2_ for 4 h at 37 °C in the CO_2_ incubator. The cell viability assay was the same as the one described above.

### 2.6. Statistical Analysis

All data shown are means of three wells. Quantitative data are expressed as the mean ± standard deviation (SD). All statistical analyses were performed in Microsoft Excel 2016 (Microsoft, Redmond, WA, USA) and Origin 8.0 (OriginLab, Northampton, MA, USA).

## 3. Results

In this paper, biological properties of new sulphur- and selenium-containing analogues of potassium phenosan were studied, and theiractivity wascompared with that of the starting hydrophobic acids in an in vitro model.

### 3.1. Synthesis and Characterization of Compounds

**Figure 1:**
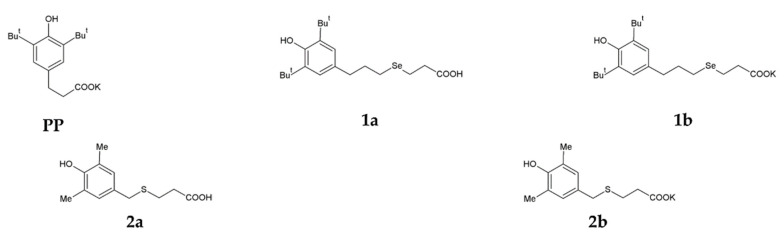
Structural formulas of potassium phenosan [**PP**, potassium 3-(3,5-di-tert-butyl-4-hydroxyphenyl)propionate], 3-[3-(3,5-di-tert-butyl-4-hydroxyphenyl)propylseleno]propanoic acid (**1a**), potassium 3-[3-(3,5-di-tert-butyl-4-hydroxyphenyl)propylseleno]propionate (**1b**), 2-(3,5-dimethyl-4-hydroxybenzylthio)propanoic acid (**2a**) and potassium 3-[4-hydroxy-3,5-dimethylbenzylthio]propanoate (**2b**).

A selenium-containing analogue of phenosanacid, 3-[3-(3,5-di-tert-butyl-4-hydroxyphenyl)propylseleno]propanoic acid (**1a**), was synthesised from bis-[3-(3,5-di-tert-butyl-4-hydroxyphenyl)propyl]diselenide by a procedure described previously [35].A sulphur-containing analogue of phenosanacid, 2-(3,5-dimethyl-4-hydroxybenzylthio)propanoic acid (**2a**), wassynthesized in reactions of 2,6-dimethylphenol with 3-thiopropanoic acid, formalin and KOH. Potassium phenosan (**PP**), and its selenium- (**1b**) and sulphur-containing (**2b**) analogues were synthesised by the reaction of the corresponding acids (phenosanacid, **1a** and **2a**) with KHCO_3_. The structure of all compounds was identified by elemental analysis and by NMR, IR and UV spectroscopy. The Materials and Methods section presents the detailed information. Structural formulas of the compounds are given in Figure 1.

### 3.2. In Vitro Cytotoxic and Cytostatic Activities

To investigate the cytotoxic and cytostatic effects of antioxidant potassium phenosan and its S- and Se-containing analogues **1b** and **2b** as well as their starting acids **1a** and **2a** on human cell lines HepG2 (hepatocellular carcinoma), Hep-2 (larynx carcinoma) and MCF-7 (breast adenocarcinoma), high-content analysis was performed. Cells were cultured for 48 h in the presence of various concentrations of a compound dissolved in ethanol, in a 50% aqueous ethanol solution or water. The cytotoxicity assay was carried out using dual staining with Hoechst 33,342 and propidium iodide (PI), and the cells were categorised as live, dead or apoptotic (Figure S7). The cytotoxic activity was determined as LC_50_ (concentration that reduces the number of live cells by 50%), and the cytostatic activity as IC_50_ (concentration that reduces the cell count by 50%). The survival curves are given in Figure S8. The LC_50_ values after incubation of the cell lines with the tested compounds for 48 h are listed in Table 1, and the IC_50_ values are presented in Table 2.

As displayed in Figure S8 and Table 1, potassium phenosan is toxic only in the millimolar range. HepG2 cells turned out to be more sensitive to potassium phenosan than did Hep-2 and MCF-7 cells (LC_50_ values differ almost 1.5- and 2-fold, respectively), and a concentration of 250 μM already influences cell growth as compared to the control.

As presented in Table 2, IC_50_ for HepG2 cells is two- and three-fold less than that for cell lines Hep-2 and MCF-7, respectively. Selenium compounds were found to be the most active, especially against the Hep-2 cell line.

Thus, the results indicate that LC_50_ and IC_50_ values were in the micromolar concentration range for all cell lines. Cytotoxic activities of selenium-containing acid **1a** and salt **1b** were comparable, whereas the cytostatic activity of salt **1b** was higher than that of acid **1a**. Sulphur compounds proved to be the least active, with the cytostatic effect being more pronounced than the cytotoxic one. The activities of salt **2b** and acid **2a** were comparable for each cell line except for MCF-7 cells, which turned out to be less sensitive to acid **2a**.

### 3.3. An Antioxidant Activity Assay

#### 3.3.1. A Ferric-Ion-Based Total Antioxidant Capacity (TAC) Assay

Antioxidant activities of potassium phenosan, of its S- and Se-containing analogues **1b** and **2b** and of ascorbic acid as a reference were measured by the 1,10-phenanthroline (o-phen) method [37] and were detectable in all the compounds (Table 3 and Figure S9). The method is based on the ability of an antioxidant to reduce the Fe(III)–phen complex to the coloured Fe(II)–phen complex at low pH (~3.6). Absorbance of the Fe(II)–phen complex was detected at 510 nm. Higher absorbance indicates higher ferric-ion-reducing power. TAC values were derived from a calibration curve of ferrous sulphate (FeSO_4_·7H_2_O) standards (20–100 μM).

Compound **2b** evidenced significantly higher ferric-ion-reducing antioxidant activity than potassium phenosan and **1b** and was comparable to ascorbic acid (**1b** ≈ **PP** < **2b** ≈ ascorbic acid). This finding indicates that **2b** possesses higher reducing capacity than potassium phenosan and **1b**. Ascorbic acid, a two-electron reductant, has a stoichiometric factor of 2 (one ascorbic-acid molecule is equivalent to two Fe(II) ions) [37]. It can be inferred that compound **2b** is also a two-electron reducing agent, whereas compounds **1b** and **PP** are one-electron reductants (Figure S9).

#### 3.3.2. The DPPH˙ and ABTS˙^+^ Radical-Scavenging Assay

Antioxidant activities of potassium phenosan, of its S- and Se-containing analogues **1b** and **2b** and of ascorbic acid as a reference compound were evaluated in a series of in vitro assays involving the DPPH˙ (2,2-di[4-tertoctylphenyl]-1-picrylhydrazyl) radical and the ABTS˙^+^ [2,2′-azino-bis-(3-ethylbenzothiazoline-6-sulphonic acid)] cation radical. The determined radical-scavenging activities (*RSAs*) are given in Figure S10. IC_50_ (concentration of a compound reducing radical concentration by 50%) values of all samples are summarised in Table 4.

The model based on the scavenging of stable DPPH˙ free radicals is considered an appropriate assay of the antioxidant property of compounds. The stable free radical has the advantage of being unaffected by side reactions, such as metal chelation and enzyme inhibition, brought about by additives [40]. According to the DPPH˙ assay, compound **2b**, which showed the lowest IC_50_, possesses the strongest antioxidant activity against the DPPH radical, being second only to ascorbic acid: ascorbic acid > **2b** > **1b** ≈ **PP** (Table 4).

ABTS˙^+^ is also a free and stable radical and is a cation too. This radical is reactive towards most antioxidants such as phenols, thiols or compounds that can give away a hydrogen atom or an electron. In the ABTS˙^+^ assay (1 mM), *RSA* values at various concentrations could be ranked as follows: **1b** > **2b** > **PP** > ascorbic acid (Figure S10b).

#### 3.3.3. Antioxidant Activity against H_2_O_2_-Induced Cytotoxicity

To investigate the antioxidant activity of potassium phenosan and its S- and Se-containing analogues **1b** and **2b** against H_2_O_2_-induced cytotoxicity to human cell lines (HepG2, Hep-2 and MCF-7), high-content analysis was performed. The concentrations of H_2_O_2_ that reduced the viability of cells by 20% and 40% were selected beforehand. Figure S11 presents the dose–response curves that helped to choose the two concentrations for each cell line that were used throughout the rest of the study.

Cells were cultured for 24 h in the presence of various concentrations of each salt (1–75 µM) that do not affect cell viability when dissolved in water. After that, the cells were exposed to H_2_O_2_ for 4 h at 37 °C in the CO_2_ incubator. Cell viability was assessed by dual staining with Hoechst 33,342 and PI, and the cells were categorised as live, dead or apoptotic.

Figure S12 shows the curves of cell survival after 24 h of exposure to a test compound without H_2_O_2_ treatment. In the concentration range from 1 to 75 μM, the compounds had no effect on cell growth and viability.

**Figure 2:**
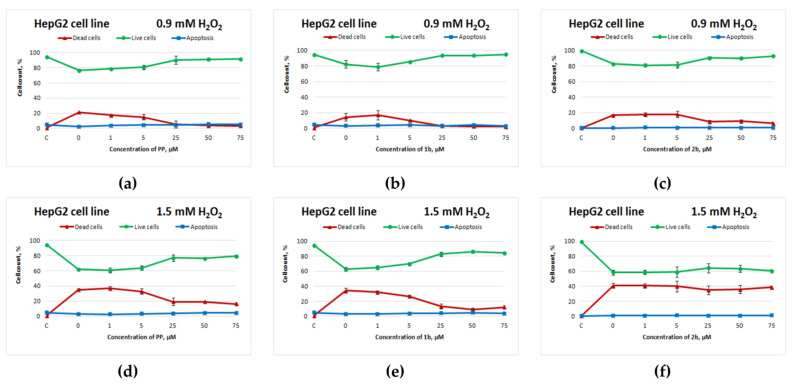
Effects of potassium phenosan [**PP**, potassium salt of potassium 3-(3,5-di-tert-butyl-4-hydroxyphenyl)propionate], potassium 3-[3-(3,5-di-tert-butyl-4-hydroxyphenyl)propylseleno]propionate (**1b**) or potassium 3-[4-hydroxy-3,5-dimethylbenzylthio]propanoate (**2b**) on the viability of HepG2 cells under the influence of H_2_O_2_ as determined by dual staining with Hoechst 33342/propidium iodide. Cells were cultured in the presence of each salt for 24 h, after which they were exposed to H_2_O_2_ for 4 h. Point C represents control cells without treatment with hydrogen peroxide. (**a**–**c**)—effect of **PP**, **1b** and **2b** on the viability of tumour cells under the influence of 0.9 mM H_2_O_2_, respectively, (**d**–**f**)—effect of **PP**, **1b** and **2b** on the viability of tumour cells under the influence of 1.5 mM H_2_O_2_, respectively.

Figure 2 depicts the curves of HepG2 cell survival after exposure to the test compounds and subsequent H_2_O_2_ treatment for 4 h.

Selenium-containing salt **1b** exerted an antioxidant action on HepG2 cells (Figure 2b,e) that was comparable to the action of potassium phenosan (Figure 2a,d) at 0.9 and 1.5 mM H_2_O_2_ (starting from 25 μM, the percentage of live cells was higher as compared to the control without compounds). Sulphur-containing salt **2b** had a positive effect on cell viability only when the cells were exposed to 0.9 mM H_2_O_2_ (Figure 2c).

**Figure 3:**
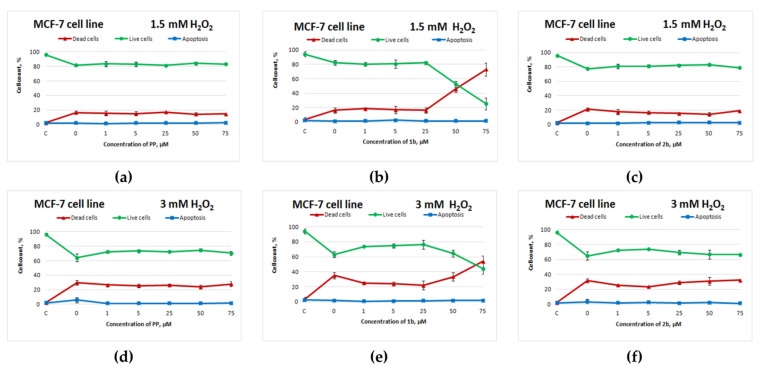
Effects of potassium phenosan [**PP**, potassium 3-(3,5-di-tert-butyl-4-hydroxyphenyl)propionate], potassium 3-[3-(3,5-di-tert-butyl-4-hydroxyphenyl)propylseleno]propionate (**1b**) or potassium 3-[4-hydroxy-3,5-dimethylbenzylthio]propanoate (**2b**) on the viability of MCF-7 cells under the influence of H_2_O_2_ as determined by dual staining with Hoechst 33342/propidium iodide. Cells were cultured in the presence of each salt for 24 h, after which they were exposed to H_2_O_2_ for 4 h. Point C represents control cells without treatment with hydrogen peroxide. (**a**–**c**)—effect of **PP**, **1b** and **2b** on the viability of tumour cells under the influence of 1.5 mM H_2_O_2_, respectively, (**d**–**f**)—effect of **PP**, **1b** and **2b** on the viability of tumour cells under the influence of 3 mM H_2_O_2_, respectively.

Potassium phenosan and **2b** did not show an antioxidant activity against MCF-7 cells (Figure 3a,d,c,f). Compound **1b**, on the other hand, exerted an antioxidant action on MCF-7 cells in the concentration range from 1 to 25 μM, but starting from 50 μM, cell death sharply increased and significantly exceeded cell death in the control with H_2_O_2_ (Figure 3b,e). Without exposure to H_2_O_2_, this negative effect of 50 μM **1b** on cell viability was absent (Figure S6h).

**Figure 4:**
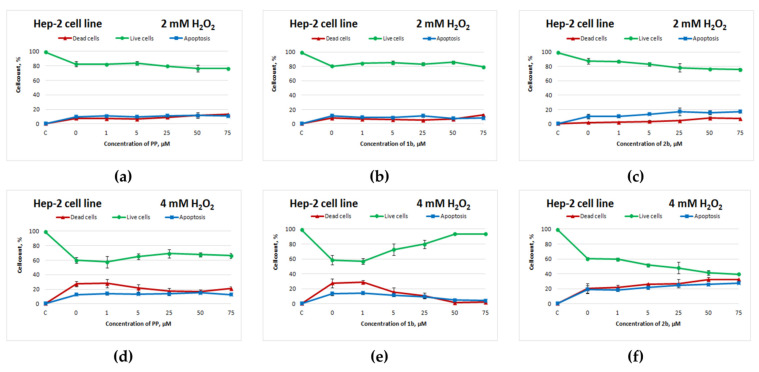
Effects of potassium phenosan [**PP**, potassium 3-(3,5-di-tert-butyl-4-hydroxyphenyl)propionate], potassium 3-[3-(3,5-di-tert-butyl-4-hydroxyphenyl)propylseleno]propionate (**1b**) or potassium 3-[4-hydroxy-3,5-dimethylbenzylthio]propanoate (**2b**) on the viability of Hep2 cells under the influence of H_2_O_2_ as determined by dual staining with Hoechst 33342/propidium iodide. Cells were cultured in the presence of each salt for 24 h, after which they were exposed to H_2_O_2_ for 4 h. Point C represents control cells without treatment with hydrogen peroxide. (**a**–**c**)—effect of **PP**, **1b** and **2b** on the viability of tumour cells under the influence of 2 mM H_2_O_2_, respectively, (**d**–**f**)—effect of **PP**, **1b** and **2b** on the viability of tumour cells under the influence of 4 mM H_2_O_2_, respectively.

The sulphur-containing analogue of potassium phenosan(**2b**) had a similar negative effect on the Hep-2 cell line: starting from 5 μM, cell death increased (Figure 4d,f). Compound **1b** manifested an antioxidant activity toward Hep-2 cells that was superior to that of potassium phenosan (Figure 4d,e).

## 4. Discussion

Phenosanacid is a hydrophobic phenolic antioxidant with pronounced biological activity, including anticancer activity [41]. The S- and Se-containing analogues of phenosanacid can exceed phenosan in the antioxidant activity owing to the presence of S and Segroups, as demonstrated successfully for phenolic compounds containing sulphide groups [42]. To create a water-soluble antioxidant, a phenosanacid potassium salt was synthesized (potassium phenosan), which, just as phenosanacid, is a promising pharmacologically active substance for use in biology and medicinal chemistry [43,44]. In this regard, the reaction of hydrophobic acids with KHCO_3_ synthesises the corresponding hydrophilic salts: Se- and S-containing analogues of potassium phenosan [35].

This paper presents astudy on biological properties of Se- and S-containing analogues of phenosanacid and potassium phenosan by analogy with hydrophobic phenosanacid with its hydrophilic derivative: potassium phenosan.

Selenium- and sulphur-containing analogues of potassium phenosan (**1b** and **2b**) are hydrophilic compounds, unlike their starting acids (**1a** and **2a**). The study on cytotoxic and cytostatic activities of S- and Se-containing compounds **1b** and **2b** and a comparison of these compounds’ properties with their starting acids **1a** and **2a** were performedby means of human cell lines HepG2 (hepatocellular carcinoma), Hep-2 (larynx carcinoma) and MCF-7 (breast adenocarcinoma). Selenium compounds were found to be the most active, especially against Hep-2 cells. The cytostatic activity of selenium-containing salt **1b** proved to be higher than that of acid **1a**. The cytostatic effects were comparable between sulphur-containing compounds **2b** and **2a**, as were cytotoxicity levels between the acids and salts. Overall, the influence of the salts on cell viability can be presented in the following order: **1b** > **PP** > **2b**.

The antioxidant activity of salts **PP**, **1b** and **2b** was evaluated using ferric-ion-based TAC, DPPH, and ABTS assays.The ferric-ion-based TAC assayshowed that sulphur compound **2b** possesses reducing capacity comparable to that of ascorbic acid and higher than reducing capacity of selenium compound **1b** and of **PP**. Ascorbic acid is a two-electron reductant [37]. It can be inferred that compound **2b** is also a two-electron reducing agent, whereas compounds **1b** and **PP** are one-electron reductants.

The reactions of antioxidants with DPPH are based on electron transfer; hence, the results overall matched the results from the ferric-ion-based TAC assay.According to the DPPH˙ assay, compound **2b** has the strongest antioxidant activity compared to **PP** and **1b**, albeit weaker than that of ascorbic acid.The salts show stronger radical-scavenging activity towards ABTS˙^+^ than towards the DPPH˙ radical. In the ABTS˙^+^ assay, the activity of compounds **PP** and **2b** was comparable and inferior to that of compound **1b**. The inhibition of ABTS˙^+^ activity in an antioxidant sample strongly correlated with DPPH˙radical-scavenging capacity because both radicals can accept electrons and H˙ from the antioxidant compounds present in the samples. The activity differences shown by these methods can be explained by different durationsof incubation with antioxidants.

The evaluation of the antioxidant activity towards H_2_O_2_-induced oxidative stress showed that the activity of the selenium-containing phenosan analogue **1b** significantly exceeds the activities of **PP** and **2b**. Overall, the addition of antioxidants to the culture medium in the concentration range up to 100 μM did not affect cell growth and viability within 48 h according to assays of cytotoxic and cytostatic activities. At the same time, a negative effect on breast cancer cell line MCF-7 was observed after hydrogen peroxide was added: at **1b** concentrations above 25 μM, exposure to H_2_O_2_ led to massive cell death, which was greater than that during exposure to H_2_O_2_ alone (control without **1b**). For the sulphur-containing salt, we registered a similar but much weaker effect on Hep-2 cells.

The reason for the negative effect of selenium- and sulphur-containing salts on the MCF-7 and Hep-2 cells, respectively, remains unclear and probably tested compounds exerted intracellular different mechanism of action in cancer cell lines in the response to antioxidants.

According to our results, Se-containing salt **1b** has good potential to affect tumour cell metabolism owingto theability to promote reduction of Fe(III) to Fe(II) in numerous cellular metalloenzymes. This process can increase oxidative stress through the Fenton reaction, which—in combination with additional upregulation of ROS—can cause cell death. Among transition metals, Fe is known as the most important pro-oxidant in terms of lipid oxidation owing to high reactivity of this metal [45]. Fe metabolism is of particular interest for the elucidation of potential vulnerability of tumour cells to agents that alter the redox balance. Many types of cancer involve aberrant regulation of an iron exporter called ferroportin (FPN-1) [46]. It is known that FPN-1 is significantly under-expressed in breast cancer, prostate cancer, and hepatocellular-carcinoma cells compared to normal cells [47]. Alteration of metabolism intumorousMCF-7 cells by Se-containing salt **1b** can be beneficial by itself and enhance theactivity of chemotherapeutic drugs that increase the concentration of ROS.

Thus, a combination of an antitumor therapy with the Se-containing potassium phenosan analogue assessed in this work may provide therapeutic advantages in the treatment of breast cancer.

## 5. Conclusions

Herein, we investigated cytotoxic, cytostatic and antioxidant activities of potassium phenosan and its new sulphur- and selenium-containing analogues toward human larynx carcinoma (Hep-2), human hepatocellular carcinoma (HepG2) and human breast adenocarcinoma (MCF-7) cells. Selenium-containing analogue **1b** has stronger cytotoxic and cytostatic properties than do the other tested compounds. In addition, against H_2_O_2_-induced cytotoxicity, **1b** has an antioxidant effect that is comparable to the effect of potassium phenosan (in HepG2 cells) or greater than that of potassium phenosan (in Hep-2 cells). **1b** exerts an antioxidant action on MCF-7 cells in the concentration range 1–25 μM, but starting from 50 μM, cell death sharply increases and significantly exceeds cell death in the control with H_2_O_2_. The prospect for further research is to examine the effect of Se-containing salt **1b** leading to increased cell death with additional induction of ROS in tumor cells MCF-7.

## Figures and Tables

**Table 1:** The cytotoxicity (LC_50_) of compounds against HepG2, Hep-2 and MCF-7 cells. The values are the mean of three wells ± standard deviation (SD).

Compound ^1^	LC_50_^2^, μM		
	HepG2 Cells	Hep-2 Cells	MCF-7 Cells
**PP**	3899 ± 39	>5000	7342 ± 74
**1a**	338 ± 20	235 ± 18	375 ± 30
**1b**	326 ± 4	190 ± 6	459 ± 28
**2a**	5729 ± 58	13,885 ± 139	>10,000
**2b**	6347 ± 64	>10,000	>10,000

^1^**PP**: potassium phenosan; **1a**: 3-[3-(3,5-di-tert-butyl-4-hydroxyphenyl)propylseleno]propanoic acid; **1b**: potassium 3-[3-(3,5-di-tert-butyl-4-hydroxyphenyl)propylseleno]propionate; **2a**: 2-(3,5-dimethyl-4-hydroxybenzylthio)propanoic acid; **2b**: potassium 3-[4-hydroxy-3,5-dimethylbenzylthio]propanoate. ^2^ All the LC_50_ values is a statistically significant difference(Student *t*-test, *p* < 0.05) with the exception of **1a** and **1b** against HepG2 cells and **1a** against HepG2 cells and **1a** against MCF-7 cells.

**Table 2:** The cytostaticity (IC_50_) of compounds against HepG2, Hep-2 and MCF-7 cells. The values are the mean of three wells ± standard deviation (SD).

Compound ^1^	IC_50_^2^, μM		
	HepG2 Cells	Hep-2 Cells	MCF-7 Cells
**PP**	964 ± 19	1911 ± 38	2628 ± 52
**1a**	413 ± 12	224 ± 5	>500
**1b**	237 ± 2	144 ± 11	265 ± 10
**2a**	2672 ± 106	3478 ± 35	8445 ± 85
**2b**	3617 ± 72	3734 ± 37	5895 ± 59

^1^**PP**: potassium phenosan; **1a**: 3-[3-(3,5-di-tert-butyl-4-hydroxyphenyl)propylseleno]propanoic acid; **1b**: potassium 3-[3-(3,5-di-tert-butyl-4-hydroxyphenyl)propylseleno]propionate; **2a**: 2-(3,5-dimethyl-4-hydroxybenzylthio)propanoic acid; **2b**: potassium 3-[4-hydroxy-3,5-dimethylbenzylthio]propanoate. ^2^ All the LC_50_ values is a statistically significant difference (Student *t*-test, *p* < 0.05).

**Table 3:** Ferric-ion-reducing activities ofthe compounds under study. Measurements were performed in triplicate, and the data represent mean ± SD.

Compound ^1^	µmol Fe(II)/mg Compound ^2^
PP	3.9 ± 0.1
1b	3.0 ± 0.1
2b	7.2 ± 0.2
Ascorbic acid	12.2 ± 0.3

^1^**PP**: potassium phenosan; **1b**: potassium 3-[3-(3,5-di-tert-butyl-4-hydroxyphenyl)propylseleno]propionate; **2b**: potassium 3-[4-hydroxy-3,5-dimethylbenzylthio]propanoate. ^2^ Concentration of a compound having a Fe(III)–phen-reducing ability expressed in μmol of Fe(II) equivalents.

**Table 4:** In vitroantioxidant activity of the test compounds. IC_50_ (concentration of a compound reducing radical concentration by 50%) values are mean ± SD of triplicates.

**Compound ^1^**	**Radical Scavenging Activities(IC_50_^2^, μM)**	
	**DPPH˙**	**ABTS˙^+^**
**PP**	>20	6.5 ± 0.2
**1b**	>20	3.2 ± 0.1
**2b**	22.9 ± 0.6	5.5 ± 0.1
Ascorbic acid	13.6 ± 0.4	11.5 ± 0.4

^1^**PP**: potassium phenosan; **1b**: potassium 3-[3-(3,5-di-tert-butyl-4-hydroxyphenyl)propylseleno]propionate; **2b**: potassium 3-[4-hydroxy-3,5-dimethylbenzylthio]propanoate. ^2^ All the LC_50_ values is a statistically significant difference (Student *t*-test, *p*< 0.05).

## Data Availability

All data generated or analysed during this study are included in this published article and its Supplementary Information Files.

## Supplementary Materials

The following supporting information can be downloaded at: https://www.mdpi.com/article/10.3390/cimb44070216/s1. Figure S1. ^1^H-NMR spectrum of 3-[3-(3,5-di-tert-butyl-4-hydroxyphenyl)propylseleno]propanoic acid (**1****a**). Figure S2. ^13^C-NMR spectrum of 3-[3-(3,5-di-tert-butyl-4-hydroxyphenyl)propylseleno]propanoic acid (**1****a**). Figure S3. ^1^H-NMR spectrum of potassium 3-[3-(3,5-di-tert-butyl-4-hydroxyphenyl)propylseleno]propionate (**1****b**). Figure S4. ^1^H-NMR spectrum of 2-(3,5-Dimethyl-4-hydroxybenzylthio) propanoic acid (**2****a**). Figure S5. ^1^H-NMR spectrum of Potassium 3-[4-hydroxy-3,5-dimethylbenzylthio] propanoate (**2****b**). Figure S6. ^13^C-NMR spectrum of Potassium 3-[4-hydroxy-3,5-dimethylbenzylthio] propanoate (**2****b**). Figure S7. Morphological changes of Hep-2 cells after 48 h incubation with Potassium 3-[3-(3,5-di-tert-butyl-4-hydroxyphenyl) propylseleno] propionate (**1b**). Figure S8. Effect of the the investigated compounds on the viability of HepG2, Hep-2 and MCF-7 cells after 48 h of incubation. Figure S9. Linearity of total antioxidant capacity (TAC). Figure S10. The radical-scavenging activity (*RSA*%) towards DPPH˙ (**a**) and ABTS˙^+^ (**b**). Figure S11. Effect of H_2_O_2_ on the viability of HepG2 (**a**), Hep-2 (**b**) and MCF-7 (**c**) cells after 4 h of incubation. Figure S12. Effect of the investigated compounds on the viability of HepG2, Hep-2 and MCF-7 cells after 24 h of incubation.
