# Supplementary material for: Antioxidant Activity of New Sulphur- and Selenium-Containing Analogues of Potassium Phenosan against H2O2-Induced Cytotoxicity in Tumour Cells
Source: Curr Issues Mol Biol. 2022 Jul 7;44(7):3131–45. doi: 10.3390/cimb44070216 (PMC9317250; doi:10.3390/cimb44070216)
Supplement: Supplementary file 1 [file cimb-44-00216-s001.zip › cimb-1786827-supplementary.pdf]

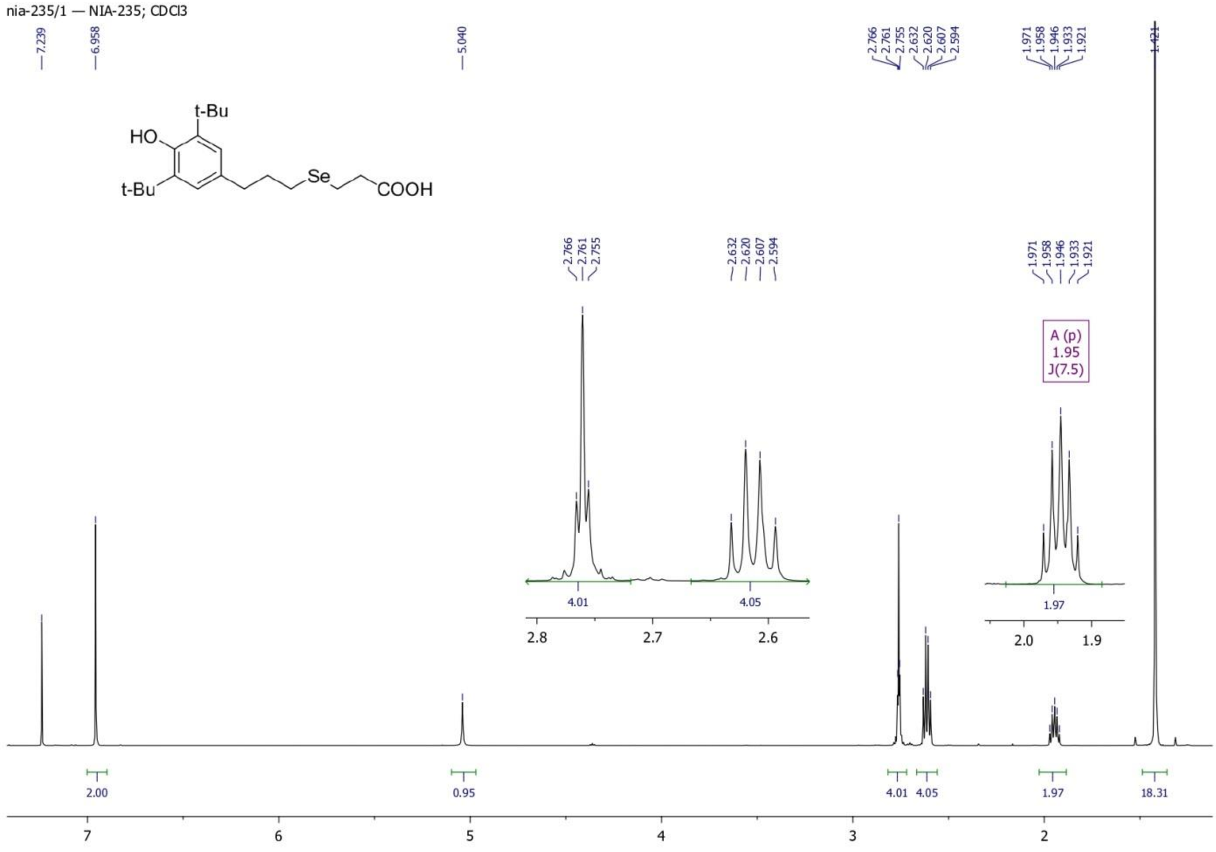


**Figure S1.** $^{1}$H-NMR spectrum of 3-[3-(3,5-di-tert-butyl-4-hydroxyphenyl)propylseleno]propanoic acid (**1a**)

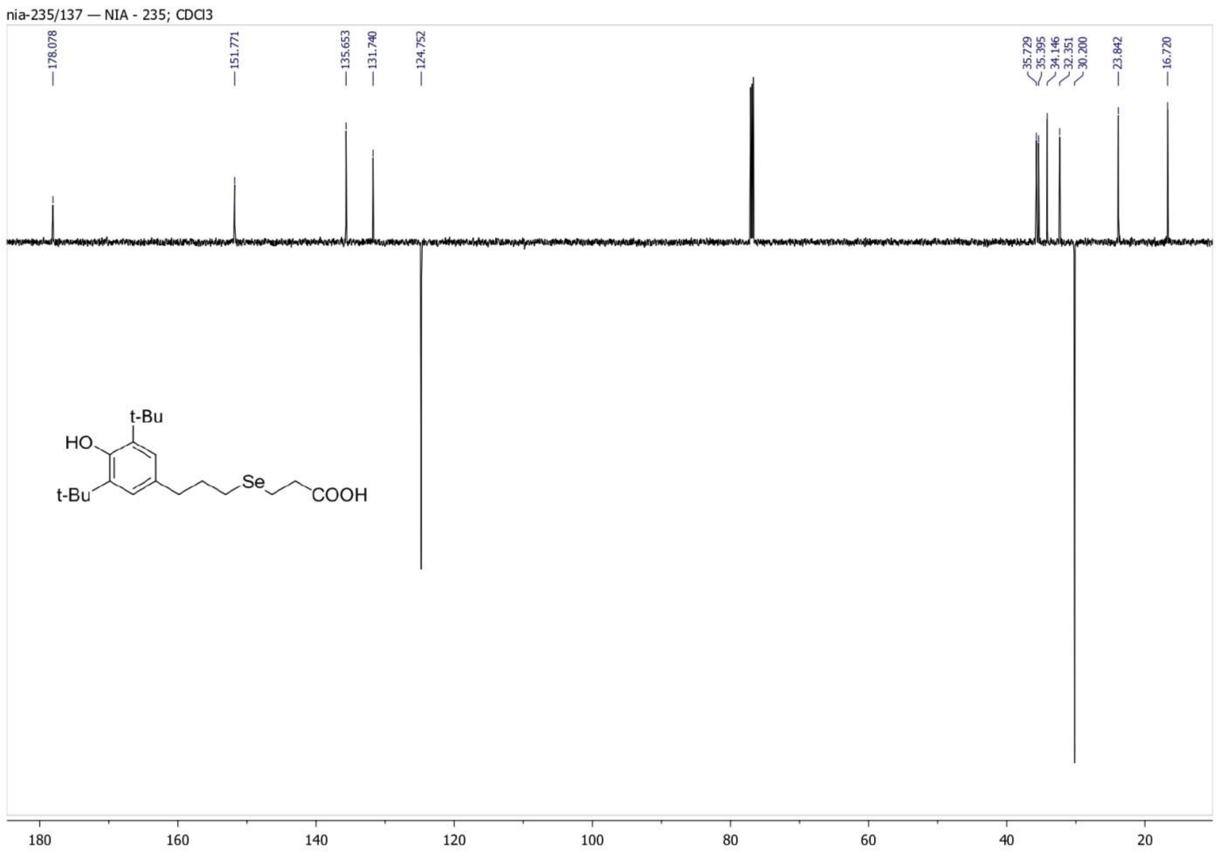


**Figure S2.** $^{13}$C-NMR spectrum of 3-[3-(3,5-di-tert-butyl-4-hydroxyphenyl)propylseleno]propanoic acid (**1a**)

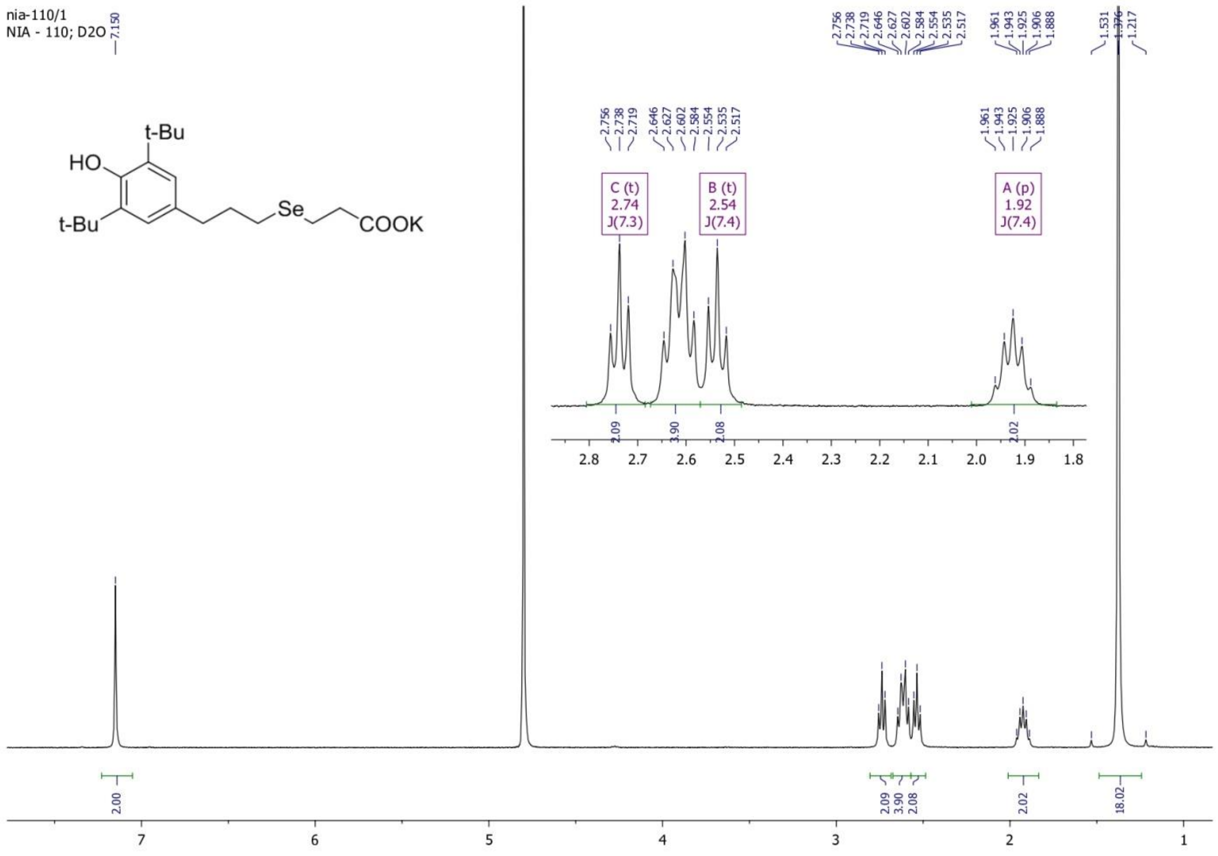


**Figure S3.** $^{1}$H-NMR spectrum of potassium 3-[3-(3,5-di-tert-butyl-4-hydroxyphenyl)propylseleno]propionate (**1b**)

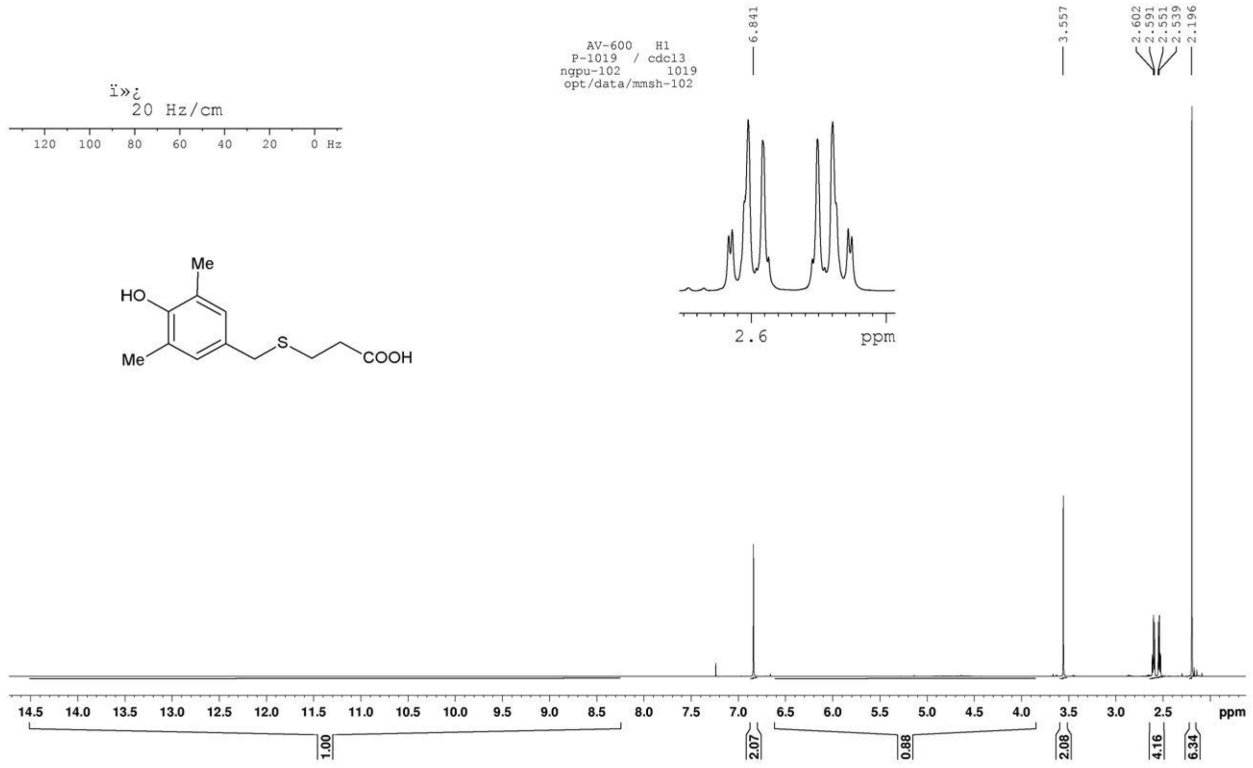


**Figure S4.** $^{1}$H-NMR spectrum of 2-(3,5-Dimethyl-4-hydroxybenzylthio) propanoic acid (**2a**)

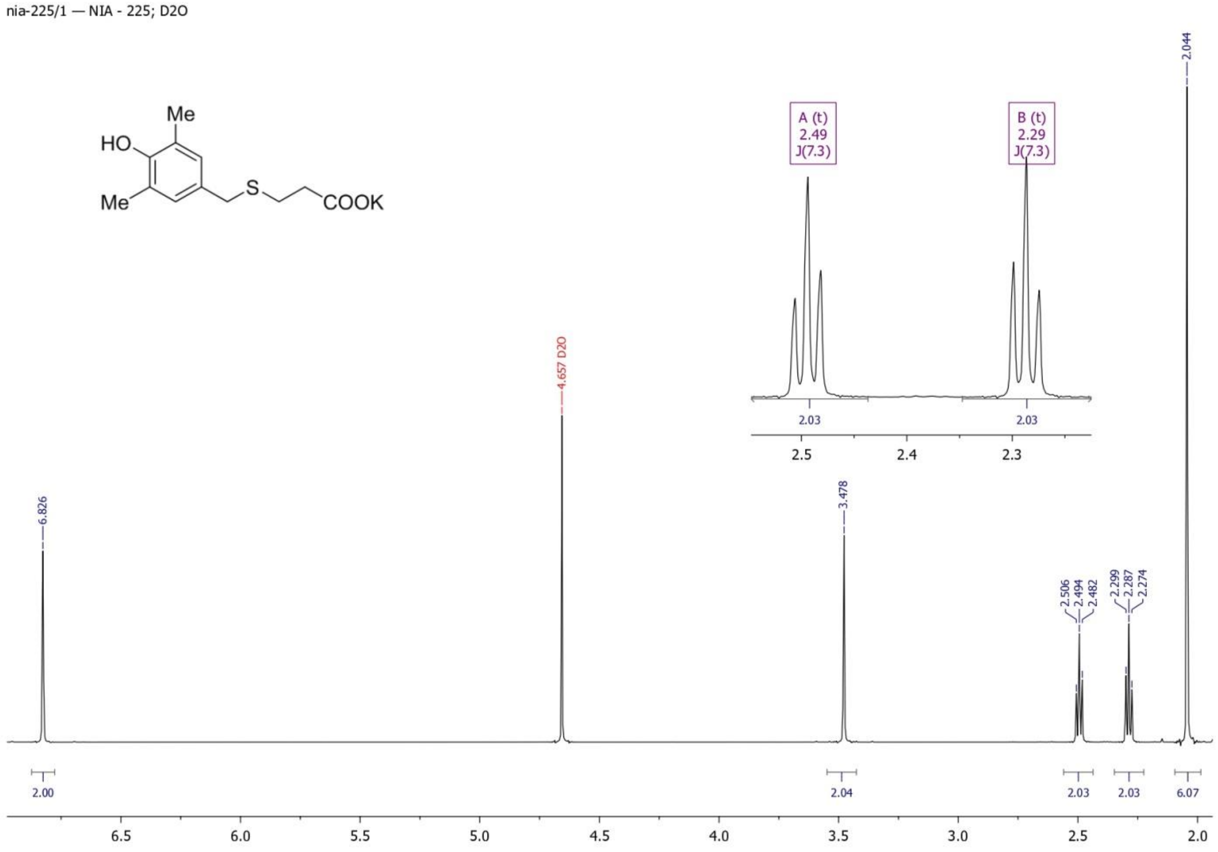


**Figure S5.** $^{1}$H-NMR spectrum of Potassium 3-[4-hydroxy-3,5-dimethylbenzylthio] propanoate (**2b**)

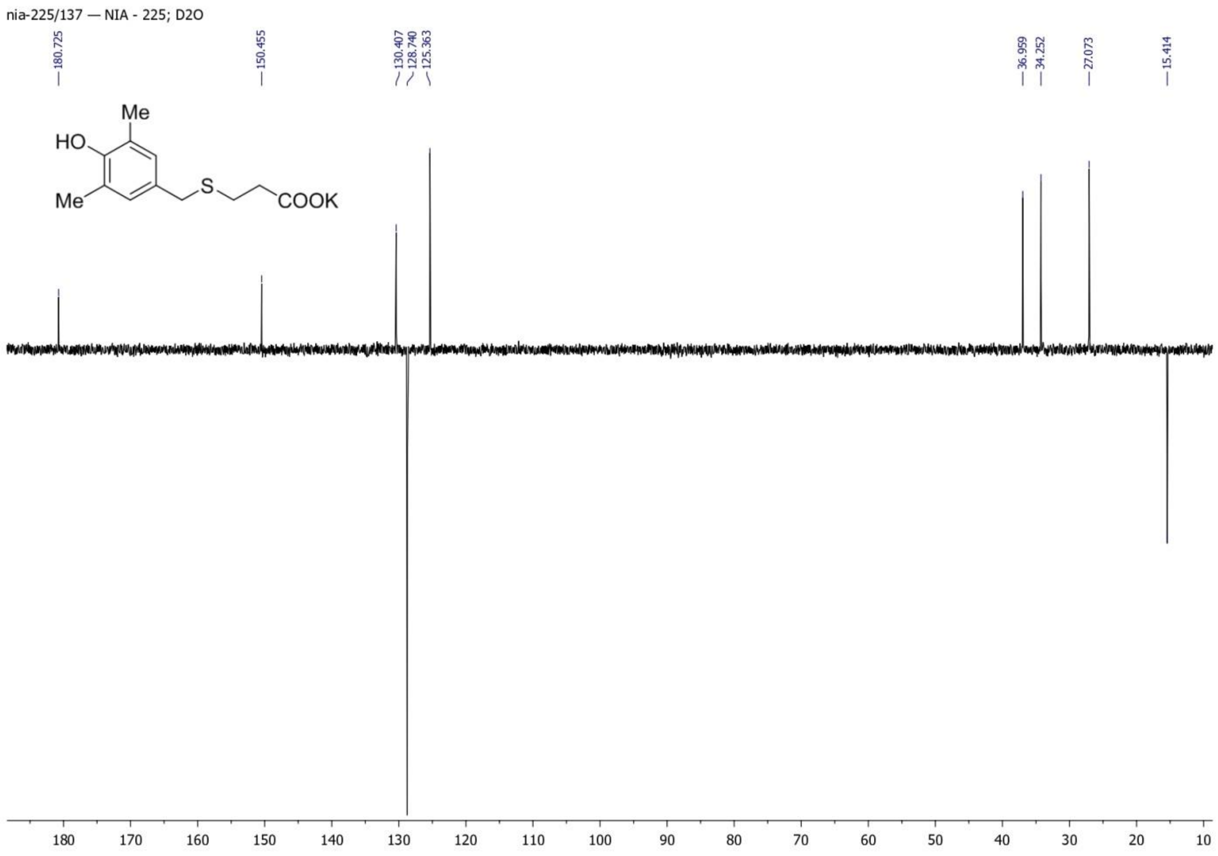


**Figure S6.** $^{13}$C-NMR spectrum of Potassium 3-[4-hydroxy-3,5-dimethylbenzylthio] propanoate (**2b**)

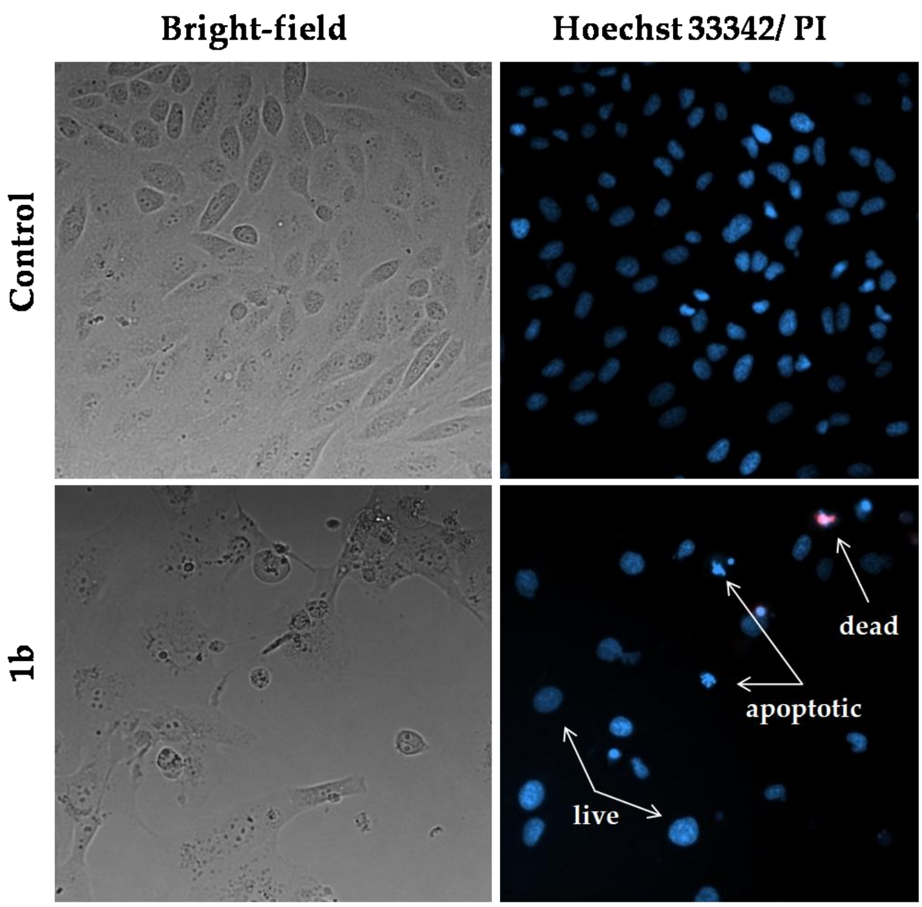


**Figure S7.** Morphological changes of Hep-2 cells after 48 hours incubation with Potassium 3-[3-(3,5-di-tert-butyl-4-hydroxyphenyl) propylseleno] propionate (**1b**), according to analysis of dual staining with Hoechst 33342/propidium iodide and bright-field microscopy. Cells were treated with: above – no treatment (control); below – **1b**, 150 μM. The cells were classified as live cells (normal nuclei: blue noncondensed chromatin uniformly dispersed over the entire nucleus), apoptotic cells (round cells, bright blue chromatin that is highly condensed or fragmented) and dead cells (red, enlarged nuclei with smooth normal structure or bright red, slightly condensed nuclei).

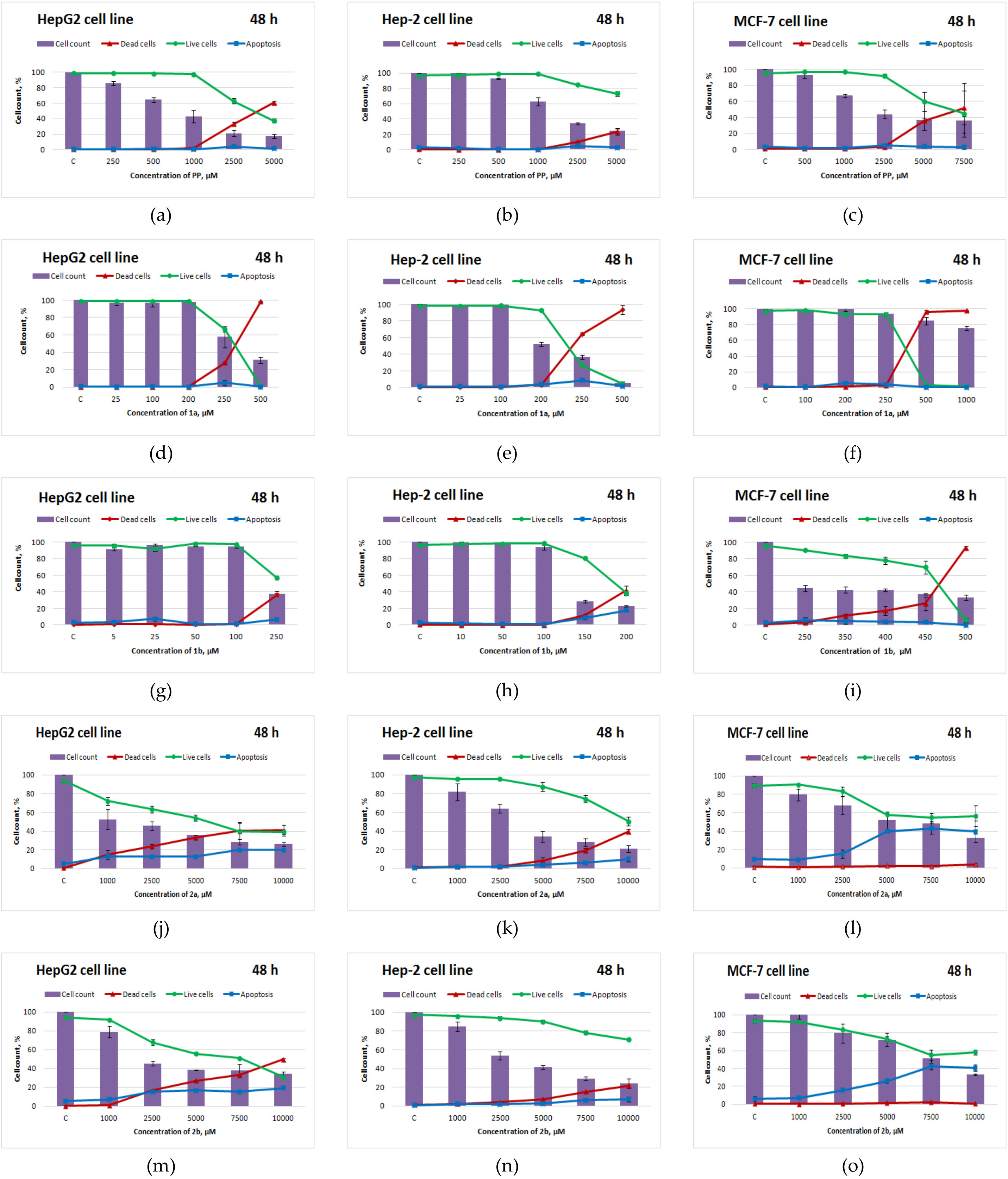


**Figure S8.** Effect of the potassium phenosan [**PP**, Potassium salt of beta-(4-hydroxy-3,5-ditretbutil-phenyl)-propionic acid], 3-[3-(3,5-di-tert-butyl-4-hydroxyphenyl) propylseleno] propanoic acid (**1a**), Potassium 3-[3-(3,5-di-tert-butyl-4-hydroxyphenyl) propylseleno] propionate (**1b**), 2-(3,5-Dimethyl-4-hydroxybenzylthio) propanoic acid (**2a**), Potassium 3-[4-hydroxy-3,5-dimethylbenzylthio] propanoate (**2b**) on the viability of HepG2, Hep-2 and MCF-7 cells after 48 hours of incubation determined by dual staining with Hoechst 33342/propidium iodide.

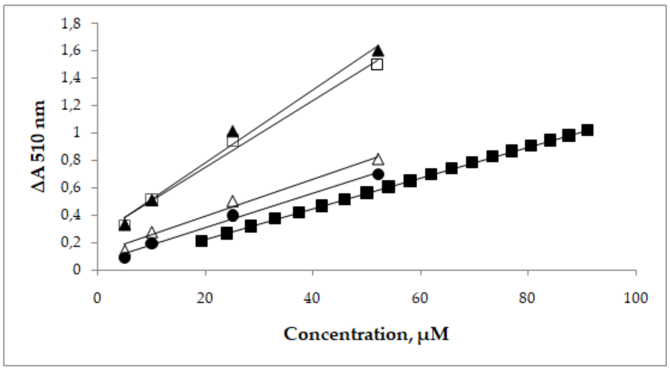


**Figure S9.** Linearity of total antioxidant capacity (TAC): dose–response lines for solutions of potassium phenosan (open triangles), the Se-containing analogue (circles), the S-containing analogue (filled triangles), ascorbic acid (open squares), and FeII (filled squares).

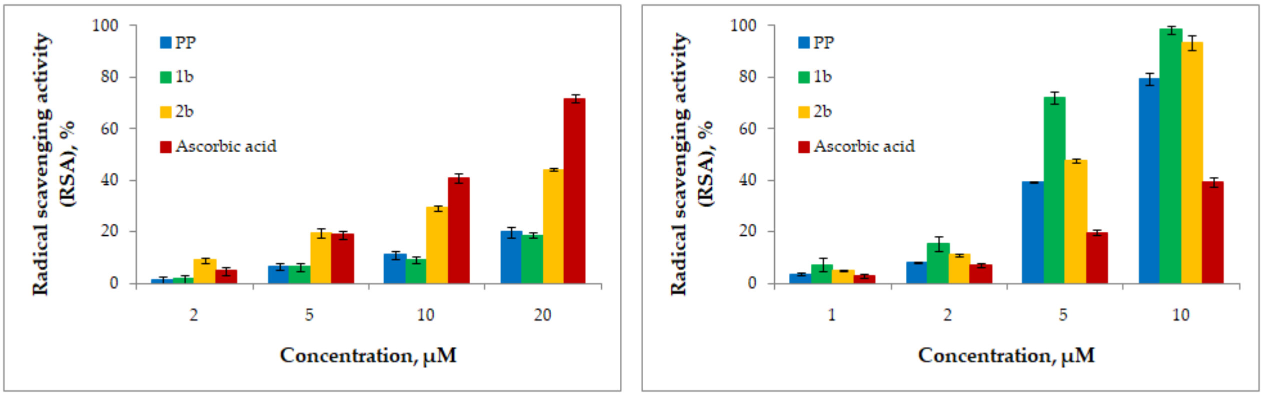


(a) (b)

**Figure S10.** The radical-scavenging activity (RSA%) towards $DPPH^{\cdot}$ **(a)** and $ABTS^{\cdot +}$ **(b)**. A comparison of different concentrations of potassium phenosan [**PP**, potassium salt of β-(4-hydroxy-3,5-ditretbutil-phenyl)-propionic acid], potassium 3-[3-(3,5-di-tert-butyl-4-hydroxyphenyl) propylseleno] propionate (**1b**), potassium 3-[4-hydroxy-3,5-dimethylbenzylthio] propanoate (**2b**) and ascorbic acid. The data are shown as means ± SD (n = 3).

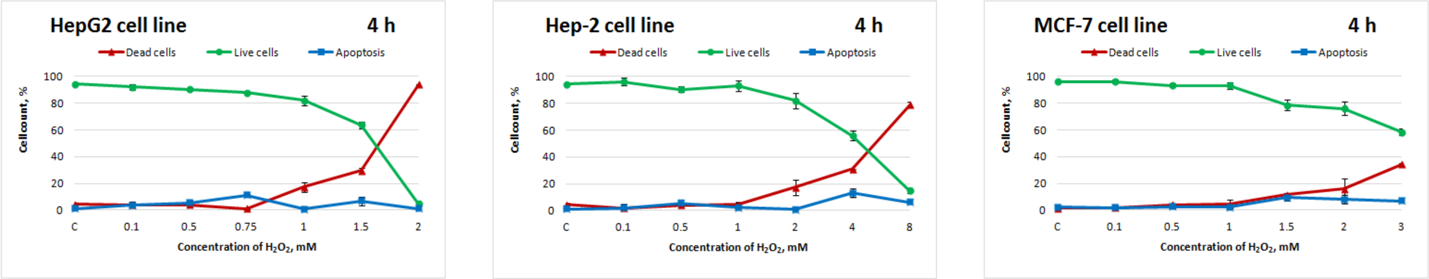


(a) (b) (c)

**Figure S11.** Effect of $H_2O_2$ on the viability of HepG2 **(a)**, Hep-2 **(b)** and MCF-7 **(c)** cells after 4 hours of incubation determined by dual staining with Hoechst 33342/propidium iodide.

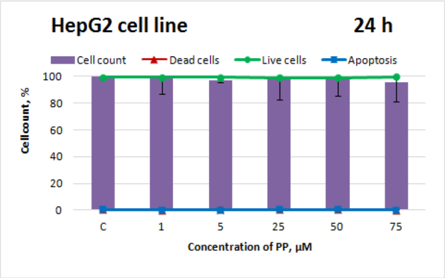


(a)

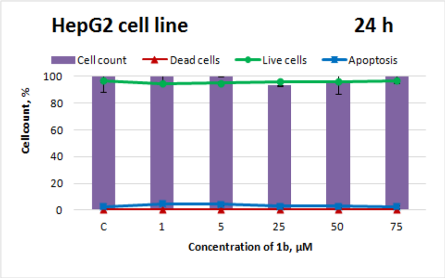


(b)

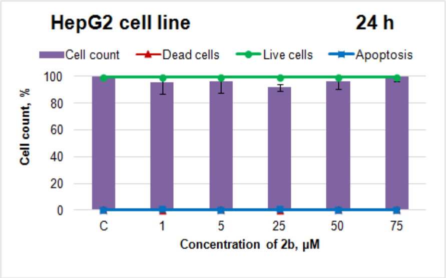


(c)

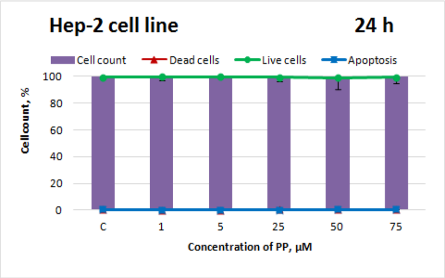


(d)

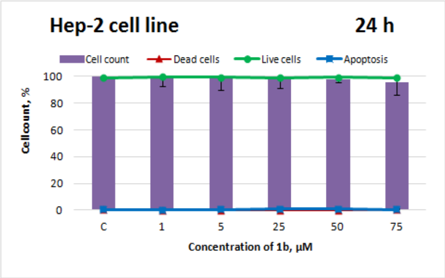


(e)

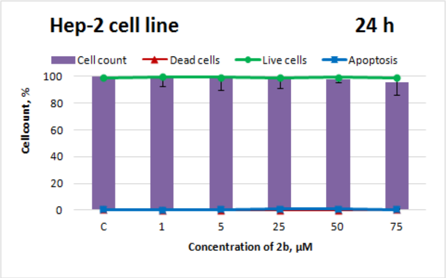


(f)

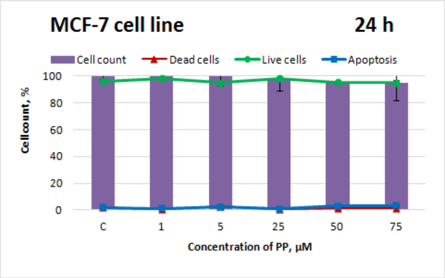


(g)

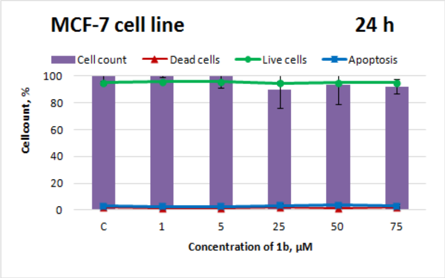


(h)

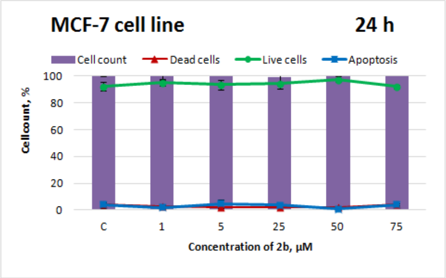


(i)

**Figure S12.** Effect of the potassium phenosan [**PP**, Potassium salt of beta-(4-hydroxy-3,5-ditretbutil-phenyl)-propionic acid], 3-[3-(3,5-di-tert-butyl-4-hydroxyphenyl) propylseleno] propanoic acid (**1a**), Potassium 3-[3-(3,5-di-tert-butyl-4-hydroxyphenyl) propylseleno] propionate (**1b**), 2-(3,5-Dimethyl-4-hydroxybenzylthio) propanoic acid (**2a**), Potassium 3-[4-hydroxy-3,5-dimethylbenzylthio] propanoate (**2b**) on the viability of HepG2, Hep-2 and MCF-7 cells after 24 hours of incubation determined by dual staining with Hoechst 33342/propidium iodide.
